# Deep learning–based reconstruction may improve non-contrast cerebral CT imaging compared to other current reconstruction algorithms

**DOI:** 10.1007/s00330-020-07668-x

**Published:** 2021-03-10

**Authors:** Luuk J. Oostveen, Frederick J. A. Meijer, Frank de Lange, Ewoud J. Smit, Sjoert A. Pegge, Stefan C. A. Steens, Martin J. van Amerongen, Mathias Prokop, Ioannis Sechopoulos

**Affiliations:** Department of Medical Imaging, Radboud University Medical Center, P.O. Box 9101 (route 766), 6500 HB Nijmegen, The Netherlands

**Keywords:** Tomography, X-ray computed, Deep learning, Brain

## Abstract

**Objectives:**

To evaluate image quality and reconstruction times of a commercial deep learning reconstruction algorithm (DLR) compared to hybrid-iterative reconstruction (Hybrid-IR) and model-based iterative reconstruction (MBIR) algorithms for cerebral non-contrast CT (NCCT).

**Methods:**

Cerebral NCCT acquisitions of 50 consecutive patients were reconstructed using DLR, Hybrid-IR and MBIR with a clinical CT system. Image quality, in terms of six subjective characteristics (noise, sharpness, grey-white matter differentiation, artefacts, natural appearance and overall image quality), was scored by five observers. As objective metrics of image quality, the noise magnitude and signal-difference-to-noise ratio (SDNR) of the grey and white matter were calculated. Mean values for the image quality characteristics scored by the observers were estimated using a general linear model to account for multiple readers. The estimated means for the reconstruction methods were pairwise compared. Calculated measures were compared using paired *t* tests.

**Results:**

For all image quality characteristics, DLR images were scored significantly higher than MBIR images. Compared to Hybrid-IR, perceived noise and grey-white matter differentiation were better with DLR, while no difference was detected for other image quality characteristics. Noise magnitude was lower for DLR compared to Hybrid-IR and MBIR (5.6, 6.4 and 6.2, respectively) and SDNR higher (2.4, 1.9 and 2.0, respectively). Reconstruction times were 27 s, 44 s and 176 s for Hybrid-IR, DLR and MBIR respectively.

**Conclusions:**

With a slight increase in reconstruction time, DLR results in lower noise and improved tissue differentiation compared to Hybrid-IR. Image quality of MBIR is significantly lower compared to DLR with much longer reconstruction times.

**Key Points:**

*• Deep learning reconstruction of cerebral non-contrast CT results in lower noise and improved tissue differentiation compared to hybrid-iterative reconstruction.*

*• Deep learning reconstruction of cerebral non-contrast CT results in better image quality in all aspects evaluated compared to model-based iterative reconstruction.*

*• Deep learning reconstruction only needs a slight increase in reconstruction time compared to hybrid-iterative reconstruction, while model-based iterative reconstruction requires considerably longer processing time.*

**Supplementary Information:**

The online version contains supplementary material available at 10.1007/s00330-020-07668-x.

## Introduction

An important factor determining image quality in computed tomography (CT) is the reconstruction algorithm. Until a decade ago, reconstruction was always performed using filtered back projection (FBP). This technique results in good image quality and is computationally very fast, but suffers from noise in low dose situations and is prone to artefacts [1]. These issues can be tackled using iterative reconstruction techniques. An iterative reconstruction technique was presented as early as in 1970 [2], but computing power limitations hindered their widespread implementation in clinical practice. It took until 2009 before the first so-called hybrid-iterative reconstruction (Hybrid-IR) methods required a low enough computing time that it allowed for widespread clinical implementation [3]. These algorithms are still based on FBP, but iteratively filter in both image and/or projection domains, resulting in both lower noise and artefacts in the reconstruction [4].

In 2011, the first full model-based iterative reconstruction (MBIR) obtained FDA clearance [3]. This reconstruction method reduces artefacts and noise even further than Hybrid-IR algorithms [5, 6]. However, their drawbacks include a higher computing power requirement, resulting in long reconstruction times, and that the reconstructed images have a plastic-like, blotchy image appearance [3, 6–8]. These factors have resulted in MBIR algorithms having a limited impact in the clinical realm.

**Fig. 1:**
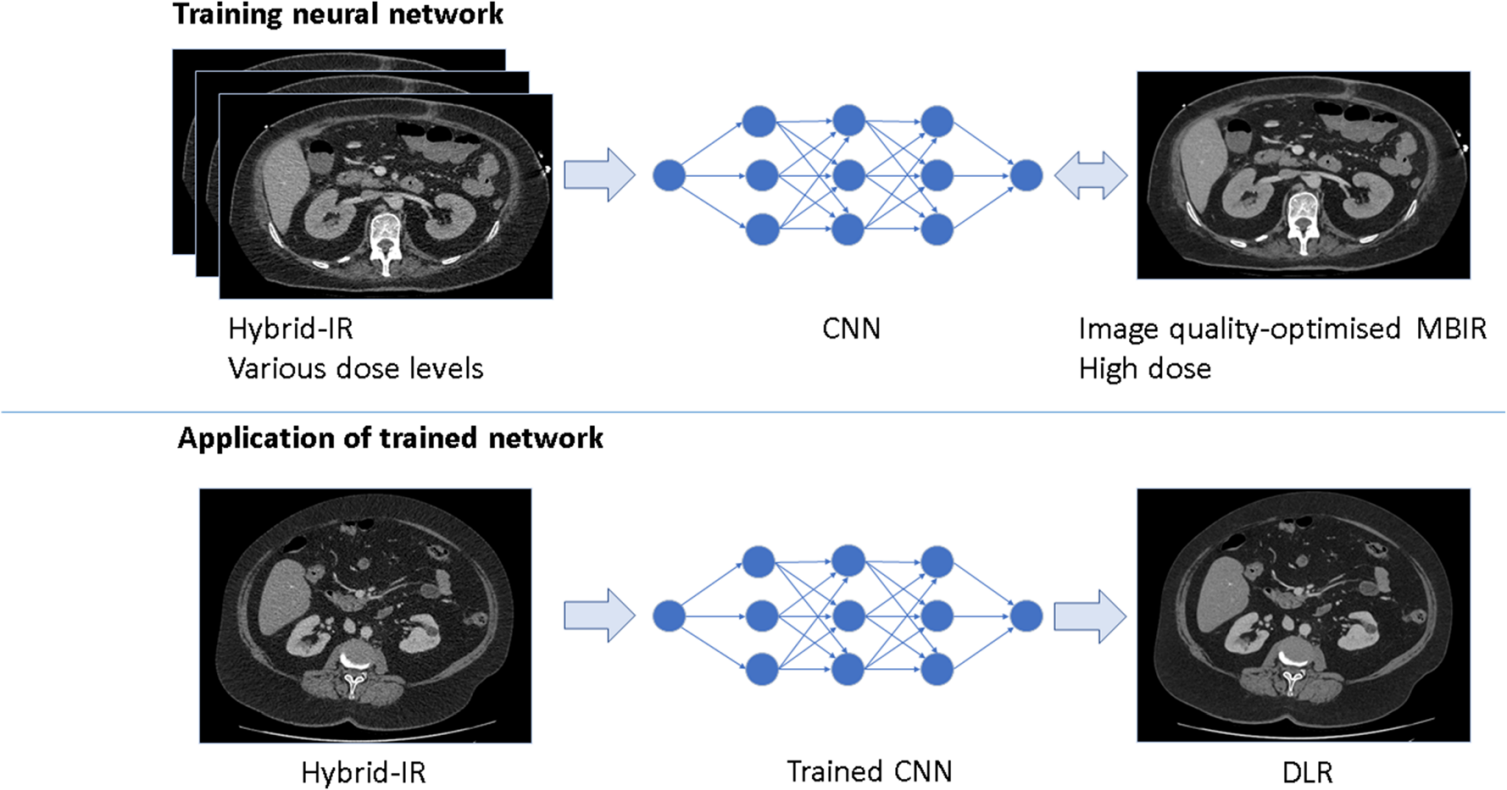
Training and application of the deep learning reconstruction (DLR) algorithm. During training, the convolutional neural network (CNN) is trained to replicate an MBIR image with the reconstruction settings set to maximise image quality, given an input Hybrid-IR image (above). In the reconstruction process, the trained network is applied to Hybrid-IR images (below)

In 2018, a new reconstruction method based on deep learning was introduced. Deep learning is used in many areas in radiology [10]. This deep learning–based reconstruction (DLR; AiCE, Canon Medical Systems Corporation) aims to reduce noise and artefacts to the same extent as MBIR, but with only a small increase in reconstruction time compared to Hybrid-IR techniques, while also resulting in a more natural, less plastic-like and blotchy, appearance than MBIR. As Fig. 1 shows, this DLR was trained on images, reconstructed from high-dose acquisitions using MBIR that is set to maximise image quality, but that takes a long time to compute. Hybrid-IR images acquired using different dose conditions were used as input. In clinical practice, the trained deep learning model is applied to Hybrid-IR images [9].

A small number of studies were published reporting on the image quality of this DLR technique applied to abdominal CT using patient and phantom images. These studies showed lower noise and higher image quality scores for DLR compared to Hybrid-IR and MBIR in both ultra-high resolution CT and conventional multi-detector CT (MDCT) [9, 11–13].

In cerebral non-contrast CT (NCCT), noise hinders the visibility of the low contrast between grey and white matter, making the promise of noise reduction with DLR of particular interest to achieve an increase in the diagnostic performance of cerebral NCCT, e.g., for improved detection of intracranial haemorrhage and of early signs of ischemia.

Therefore, the objective of this work is to evaluate the image quality and reconstruction times of DLR in comparison to Hybrid-IR and MBIR for cerebral NCCT. Our hypothesis is that the image quality resulting from DLR is comparable or superior to that from Hybrid-IR and MBIR, with a shorter image reconstruction time than that of MBIR.

## Methods and materials

An observer study was performed to assess and compare perceived image quality, and objective analysis was undertaken to quantitatively compare image signal and noise characteristics. This retrospective study was approved by the regional ethics committee (file number CMO 2016-3045, project 19051), which waived the requirement for patient informed consent after de-identification of all patient information from the study data.

### Study design and study population

Cerebral NCCT scans in a consecutive cohort of 50 patients that underwent NCCT between 14 June 2019 and 7 September 2019 for various clinical indications were collected. Two scans were incorrectly labelled and appeared to be contrast scans and were excluded. Of the remaining 48 patients, 28 were male, the age range was 19–86 years with a median age of 66 years. During clinical interpretation, intracranial haemorrhage was found in 19 patients, 13 patients had signs of infarct, and a tumour was noted in 4 patients. Seven patients had a ventricular shunt in place, four patients had a coiled aneurysm, two had an intracranial pressure monitor, and one had a stereotactic frame.

### CT acquisition and reconstruction

All acquisitions were performed on a 320 row-detector CT scanner (Aquilion One PRISM edition, Canon Medical Systems Corporation). Patients were scanned using different scan modes and techniques, as listed in Table 1.

**Table 1:** Parameters used for the non-contrast brain CT acquisitions

Parameter	Helical mode (*n* = 35)	Sequential mode (*n* = 11)	Volume mode (*n* = 2)
Tube voltage (kVp)	120	120	120
Effective tube current time product (mAs)	360	350	140/225^1^
Rotation time (s)	0.75	1.0	0.5/0.75^1^
Collimation (mm)	0.5 × 40	0.5 × 80	0.5 × 320
Pitch	0.625	n.a.	n.a.
CTDI_vol_ (mGy)	52.7	43.2/34.2^2^	16.1/25.5^1^

^1^In total, two patients were scanned using volume mode. One with a lower mAs and rotation time than the other

^2^One patient was scanned using the large field-of-view instead of the default medium field-of-view, resulting in a lower CTDI_vol_

All acquisitions were reconstructed using three reconstruction techniques: Hybrid-IR (AIDR 3D Enhanced, brain-kernel FC26), MBIR (FIRST Brain Standard), and DLR (AiCE Brain LCD). All reconstructions had a slice thickness of 0.5 mm, and the same field of view.

### Observer study and quantitative measurements

The image quality of the 48 reconstructed NCCTs was evaluated by four experienced radiologists and one final-year resident specialising in neuroradiology. Noise magnitude, sharpness, natural appearance, artefacts, grey-white matter differentiation and overall perceived image quality were scored using a 5-point Likert scale, as detailed in Table 2. The assessments were performed on a workstation with calibrated diagnostic screens (Barco MDNC-3321) in a radiology reading room with dimmed lighting. The observers were blinded to the reconstruction technique and images were presented in a random order. The order of the cases and reconstruction techniques differed for each observer.

**Table 2:** Description of the categories of image quality characteristics

Image quality characteristic	Score				
	1	2	3	4	5
Overall perceived image quality	Non-diagnostic	Poor image quality, insufficient for the evaluation of subtle pathology	Moderate image quality, sufficient for soft tissue evaluation	Good image quality; equal to current standard	Excellent image quality, superior to current standard
Natural appearance	Unnatural appearance impairing diagnostic quality	Substantial unnatural appearance, moderate impairment of diagnostic quality	Moderate unnatural appearance, not impairing diagnostic quality	Natural appearance is normal; equal to current standard	Natural appearance is superior to current standard
Artefacts	Severe artefacts, non-diagnostic quality	Substantial artefacts, moderate impairment of diagnostic quality;	Clearly visible artefacts, no impairment of diagnostic quality;	Hardly visible artefacts	No artefacts
Noise	Excessive noise, impairs diagnostic quality	Substantial noise increase; reduced image quality	Moderate increase of noise compared to current standard	Average noise; equal to current standard	Low noise magnitude, lower than current standard
Sharpness	Excessive blurring, impairs diagnostic quality	Substantial blurring; reduced image quality	Moderate sharpness; less sharpness compared to current standard	Average sharpness; equal to current standard	Sharp delineation of structures, superior to current standard
Grey-/white-matter differentiation	Poor GM/WM differentiation, impairing diagnostic quality	Reduced GM/WM differentiation, reduced diagnostic quality	Acceptable GM/WM differentiation; lower to current standard	Average GM/WM differentiation; equal to current standard	Better GM/WM differentiation compared to current standard

*GM*: grey matter; *WM*: white matter

Three ROIs were placed in each of the reconstructed volumes by an imaging scientist with 22 years of experience in x-ray imaging (L.O.), supervised by a neuro-radiologist (E.S.), encompassing different structures: cerebro-spinal fluid (CSF) within the lateral ventricle, centrum semiovale (white matter) and putamen (grey matter). The oval-shaped ROIs contained between 311 and 475 pixels. The standard deviation in the CSF (SD_CSF_) was determined as a measure of noise magnitude, and the signal-difference-to-noise ratio (SDNR) of the grey and white matter was calculated using (HU_putamen_ – HU_centrum semiovale_)/SD_CSF_, where HU is the mean of the Hounsfield Units in the voxels in the respective ROIs. Since the posterior fossa has more bony structures and therefore might be more prone to artefacts, the SD in the fourth ventricle was also measured, to determine if different values would be found compared to the measurement in the lateral ventricle.

In order to obtain a measure of the reconstruction duration, the total reconstruction time for each reconstruction method was measured manually for the first 15 acquisitions. Since the reconstructions were performed on the CT system, the reported reconstruction times are representative of those that can be expected in clinical practice.

### Statistical analysis

Mean values for the scored parameters in the observer study were estimated using a general linear model (GLM) to account for multiple readers. The full factorial GLM was built using reconstruction method and reader as factors. The estimated means for the reconstruction methods per parameter were pairwise compared and Bonferroni correction was used to adjust for multiple comparisons. Quantitative measures for the different reconstruction methods were compared using paired *t* tests.

Subgroup analysis was performed evaluating image quality of NCCT scanned in sequential and helical modes. The same method as described above was used, but a factor containing the acquisition type was added to the GLM. In order to see if artefacts appear differently across the three reconstruction methods, another subgroup analysis was performed using only scans of patients having foreign bodies.

## Results

### Observer study

**Fig. 2:**
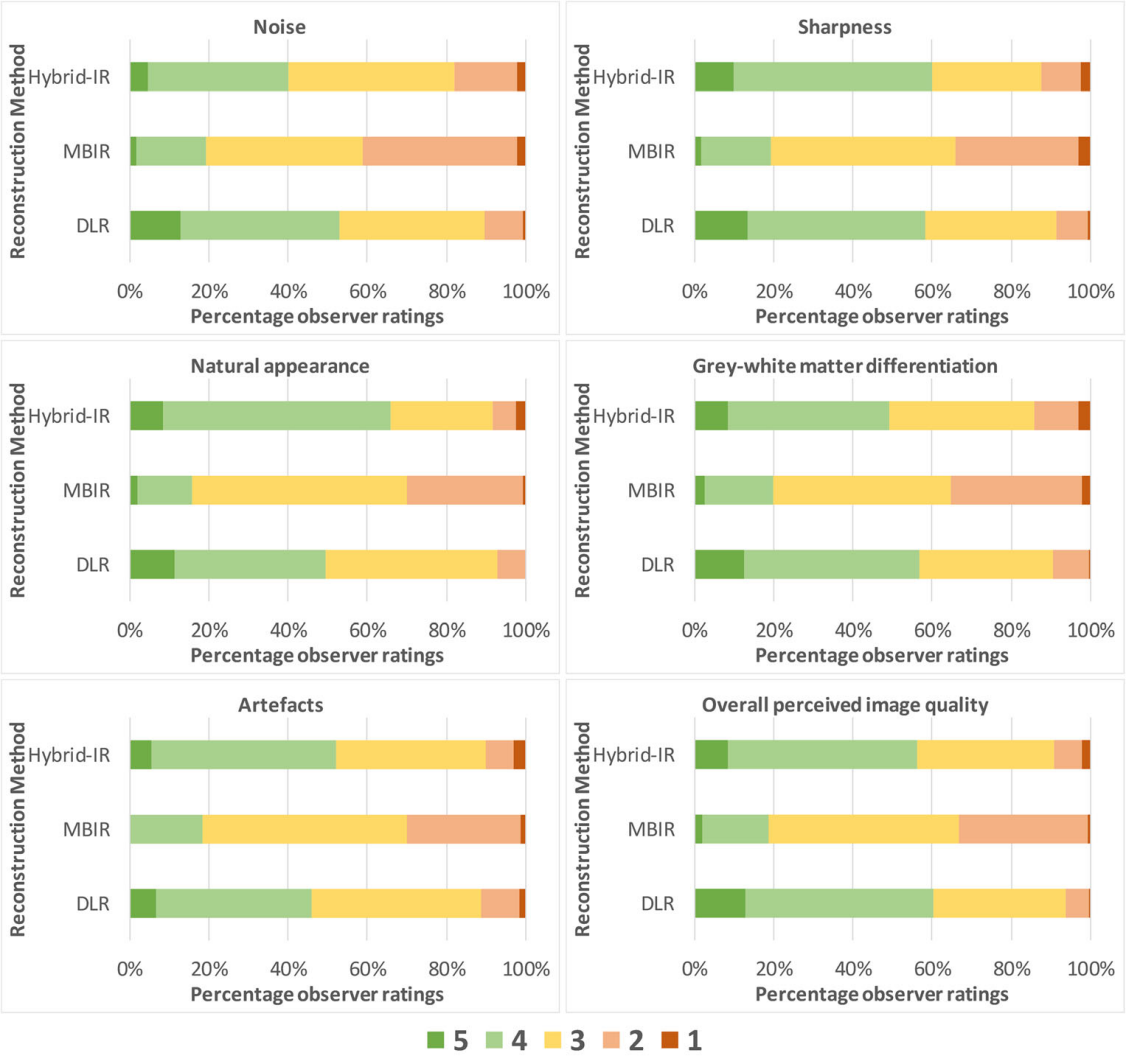
Stacked bar graph with ratings for subjective image quality criteria across all cases and observers (5-point scale; 1 = impairing diagnostic performance, 5 = better than current standard)

**Fig. 3:**
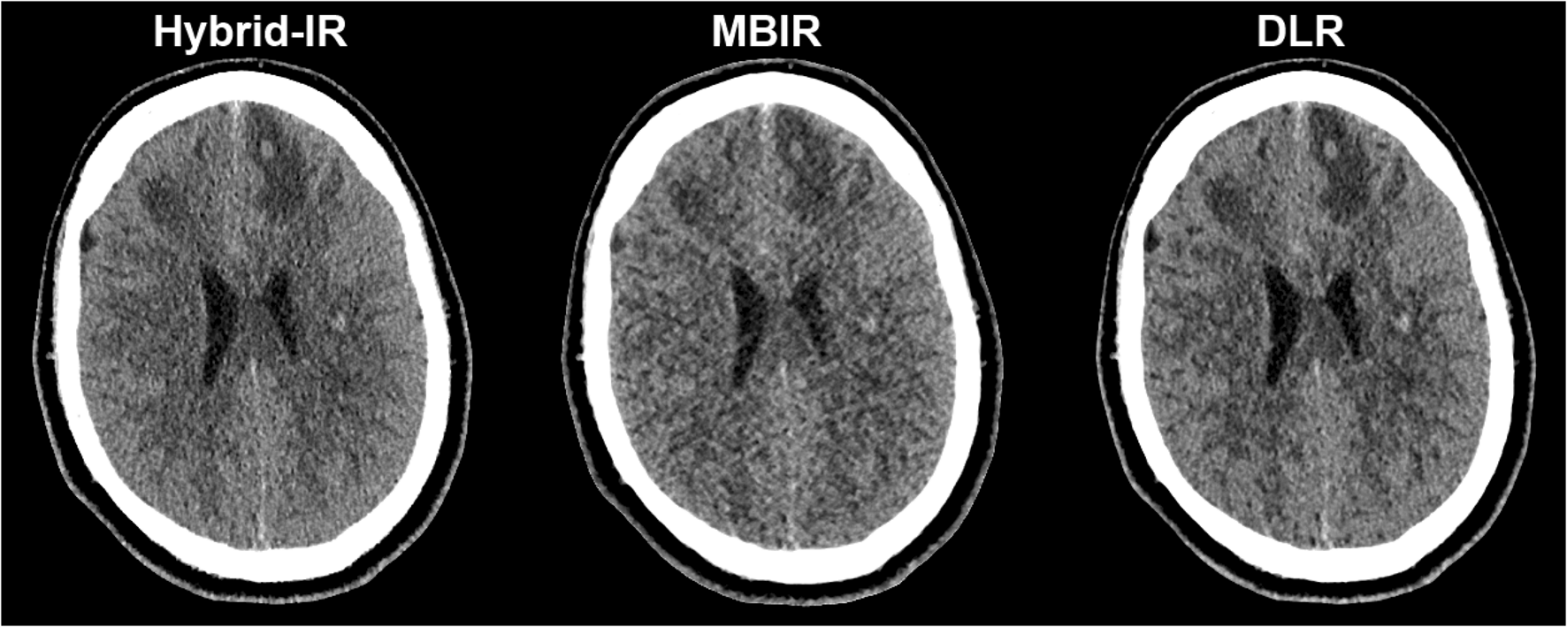
Example slice demonstrating the impact of the different reconstruction algorithms for the same non-contrast CT acquisition in a patient with small intracerebral haemorrhages with surrounding oedema

Figure 2 shows stacked bar graphs for the observer ratings of each image quality characteristic across all cases and observers. In MBIR, a maximum of 20% of the ratings was scored as 4 or 5, while for DLR at least 50% achieved these ratings. The differences in ratings for DLR and Hybrid-IR are less distinct and vary across image quality characteristics. The estimated marginal means for each reconstruction method resulting from the GLM are shown in Table 3. For all image quality characteristics, DLR scores were significantly better compared to MBIR, while compared to Hybrid-IR perceived noise and grey-white differentiation were preferred with DLR. An example slice reconstructed with the three different reconstruction algorithms is provided in Fig. 3. The image noise is notably lower for DLR as compared to Hybrid-IR and MBIR, resulting in better differentiation between grey and white matter. Table 4 shows the results found when analysing the scans of only the patients having a foreign body. The scores did not show any significant differences in the observer preferences than those found for the entire case set.

**Table 3:** Summary of results of the observer study and quantitative measurements. For the observer study, estimated marginal means for every quality parameter, reconstruction algorithm and significance versus DLR are given, with 95% confidence intervals in brackets. For the quantitative measurements, mean and standard deviation are shown

		Hybrid-IR	MBIR	DLR	DLR vs. Hybrid-IR	DLR vs. MBIR
Perceived ratings						
	Noise	3.21 [3.12–3.30]	2.78 [2.69–2.87]	3.55 [3.46–3.64]	*p* < 0.001	*p* < 0.001
	Sharpness	3.55 [3.45–3.65]	2.84 [2.74–2.94]	3.62 [3.52–3.72]	*p* = 0.947	*p* < 0.001
	Natural appearance	3.63 [3.54–3.73]	2.87 [2.78–2.96]	3.54 [3.45–3.63]	*p* = 0.446	*p* < 0.001
	Grey-white matter differentiation	3.40 [3.31–3.50]	2.85 [2.75–2.95]	3.59 [3.49–3.69]	*p* = 0.027	*p* < 0.001
	Artefacts	3.45 [3.35–3.54]	2.86 [2.76–2.96]	3.40 [3.30–3.49]	*p* = 1.000	*p* < 0.001
	Overall perceived image quality	3.53 [3.44–3.63]	2.87 [2.28–2.96]	3.67 [3.57–3.76]	*p* = 0.154	*p* < 0.001
Quantitative analysis						
	Standard deviation in lateral ventricle	6.4 ± 1.1	6.2 ± 0.7	5.6 ± 1.0	*p* < 0.001	*p* < 0.001
	Signal-difference-to-noise-ratio	1.9 ± 0.6	2.0 ± 0.6	2.4 ± 0.7	*p* < 0.001	*p* < 0.001
	Standard deviation in fourth ventricle*	6.3 ± 1.5	6.2 ± 0.7	5.3 ± 1.0	*p* < 0.001	*p* < 0.001
Reconstruction time						
	Average reconstruction time [s]	27	176	44		

*Two patients were excluded from these measurements as the fourth ventricle was filled with blood

**Table 4:** The results found when analysing the scans of only the patients having a foreign body. The scores did not show any significant differences in the observer preferences than those found for the entire case set

		Hybrid-IR	MBIR	DLR	DLR vs. Hybrid-IR	DLR vs. MBIR
Perceived ratings						
	Noise	2.87 [2.71–3.04]	2.50 [2.34–2.66]	3.26 [3.09–3.42]	*p* = 0.001	*p* < 0.001
	Sharpness	3.13 [2.95–3.30]	2.53 [2.35–2.70]	3.17 [3.00–3.35]	*p* = 0.733	*p* < 0.001
	Natural appearance	3.23 [3.06–3.40]	2.57 [2.40–2.74]	3.23 [3.06–3.40]	*p* = 1.000	*p* < 0.001
	Grey-white matter differentiation	2.94 [2.76–3.13]	2.57 [2.38–2.76]	3.33 [3.14–3.52]	*p* = 0.005	*p* < 0.001
	Artefacts	2.86 [2.68–3.03]	2.50 [2.32–2.68]	2.81 [2.64–2.99]	*p* = 0.734	*p* = 0.013
	Overall perceived image quality	3.13 [2.95–3.30]	2.53 [2.35–2.70]	3.31 [3.14–3.49]	*p* = 0.141	*p* < 0.001
Quantitative analysis						
	Standard deviation in lateral ventricle	6.6 ± 1.1	6.4 ± 0.8	5.7 ± 1.2	*p* < 0.001	*p* = 0.02
	Signal-difference-to-noise-ratio	1.8 ± 0.4	1.9 ± 0.5	2.4 ± 0.7	*p* < 0.001	*p* = 0.01
	Standard deviation in fourth ventricle*	6.2 ± 0.3	6.3 ± 1.4	5.1 ± 0.9	*p* = 0.01	*p* = 0.02

*Two patients were excluded from these measurements as the fourth ventricle was filled with blood

The ratings of the different observers are shown in Fig. S1 (supplementary online material) using stack bar graphs per observer and per image quality characteristic. Dichotomising the scores per image quality characteristic between “DLR performs equal or better” and “DLR performs worse” when comparing DLR to the other reconstruction techniques results in a maximum 10% deviation from the mean for each observer compared to the overall mean across observers, except for the mean score of one observer when assessing the grey-white matter differentiation (15%), for one observer evaluating sharpness (− 31%), and two observers when evaluating natural appearance (36% and 31%). These four larger differences were found in the comparison between DLR and Hybrid-IR. Table 5 shows that all image quality characteristics were scored higher for NCCTs scanned in sequential mode in comparison to helical mode. Using this model, which includes acquisition technique, the reconstruction method remains a significant factor (*p* < 0.001), while the interaction between reconstruction method and acquisition technique was non-significant (*p* = 0.21).

**Table 5:** Summary observer study results and quantitative measurements split to sequential and helical scanning modes. For the observer study, estimated marginal means for every quality parameter, reconstruction algorithm and significance are given, with 95% confidence intervals in brackets. For the quantitative measurements, mean and standard deviation are shown

		Sequential	Helical	*p* value
Perceived ratings				
	Noise	3.57 [3.47–3.68]	3.13 [3.08–3.19]	*p* < 0.001
	Sharpness	3.72 [3.61–3.83]	3.31 [3.24–3.37]	*p* < 0.001
	Natural appearance	3.78 [3.67–3.89]	3.33 [3.28–3.39]	*p* < 0.001
	Grey-white matter differentiation	3.75 [3.64–3.87]	3.24 [3.18–3.30]	*p* < 0.001
	Artefacts	3.77 [3.67–3.88]	3.30 [3.25–3.36]	*p* < 0.001
	Overall perceived image quality	3.41 [3.30–3.52]	3.27 [3.21–3.32]	*p* < 0.001
Quantitative analysis				
	Standard deviation in CSF	5.4 ± 0.6	6.1 ± 0.9	*p* < 0.001
	Signal-to-noise-ratio	2.4 ± 0.5	2.1 ± 0.7	*p* = 0.005

### Quantitative measurements

**Fig. 4:**
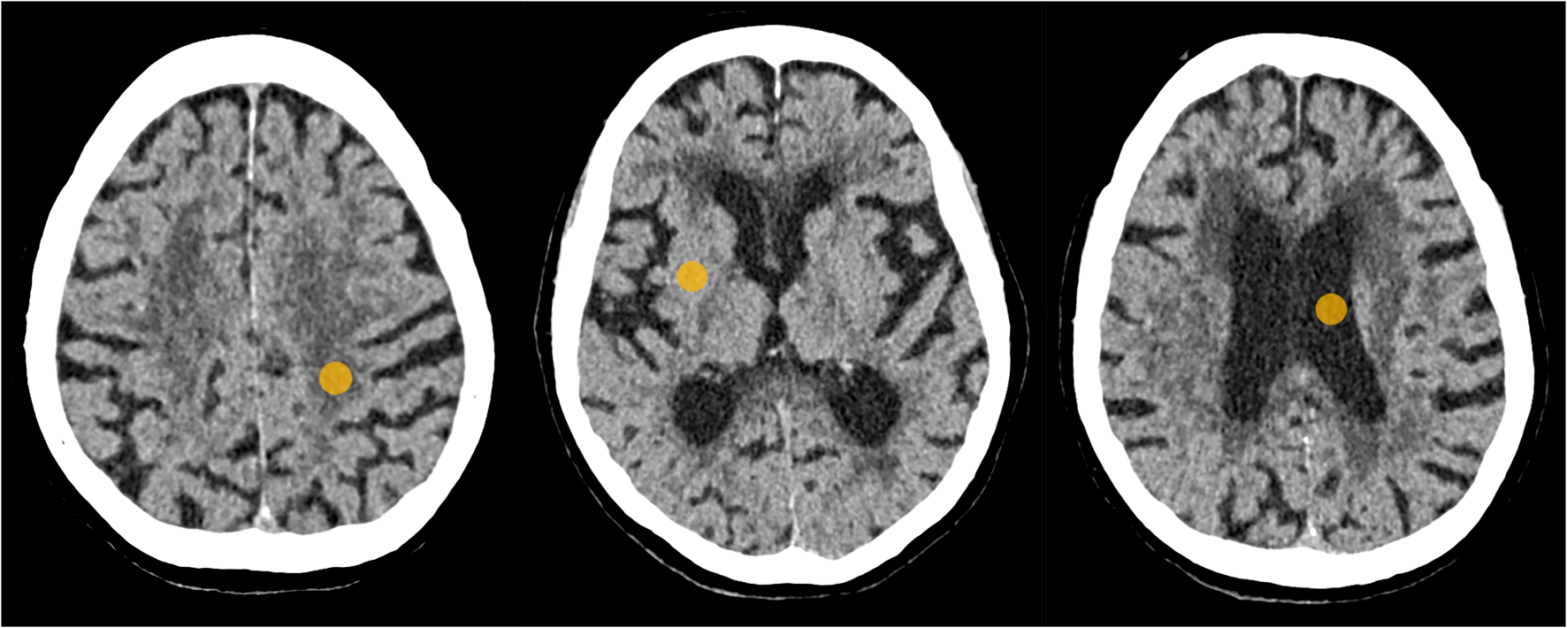
Example of the ROIs placed in the three different structures: centrum semiovale (left), putamen (middle) and cerebro-spinal fluid within the lateral ventricle (right)

The positions of the ROIs in the CSF within the lateral ventricle, centrum semiovale and putamen are shown in Fig. 4. The measured noise magnitude (SD_csf_) of DLR was clearly lower than the noise magnitude for Hybrid-IR and MBIR, as can be seen in Table 3. DLR also resulted in the highest SDNR between grey and white matter. These improvements in image quality are obtained with a modest increase in reconstruction time compared to Hybrid-IR (+ 17 s), but with substantial reduction in reconstruction time compared to MBIR (− 132 s). The SD_csf_ and SDNR were significantly better for sequential scans than those of the helical scans, as shown in Table 5.

## Discussion

This study evaluated the image quality in cerebral NCCT resulting from DLR, and it was determined that with a small reconstruction time penalty, DLR results in improved noise and tissue differentiation compared to Hybrid-IR, while maintaining the quality of the other quality characteristics. For this, DLR needs 17 s extra reconstruction time. DLR performs better for all image quality characteristics compared to MBIR. These results are in line with studies evaluating DLR in body applications [9, 12–14]. These studies showed a lower noise and a higher SDNR for DLR compared to Hybrid-IR and MBIR, although these studies also showed a higher perceived image quality score. In this study, a statistically significant difference in the overall quality rating between DLR and Hybrid-IR was not detected, although in a majority of cases the perceived image quality of DLR was rated higher than Hybrid-IR.

The lower measured noise and therefore the higher SDNR might allow for lowering the radiation dose. The noise in DLR images is about 1.3 times lower than that in resulting from Hybrid-IR. Although noise decreases with the square root of the dose in filtered back projection, this is not the case for DLR and Hybrid-IR [11, 15], making it impossible to directly determine what dose reduction could be achieved while maintaining the SDNR, based solely on the results of this study. For this, further investigation on noise characteristics, magnitude and texture, as function of dose, would be needed. Of course, another alternative is to maintain the same dose levels, which could result in improved diagnostic performance.

**Fig. 5:**
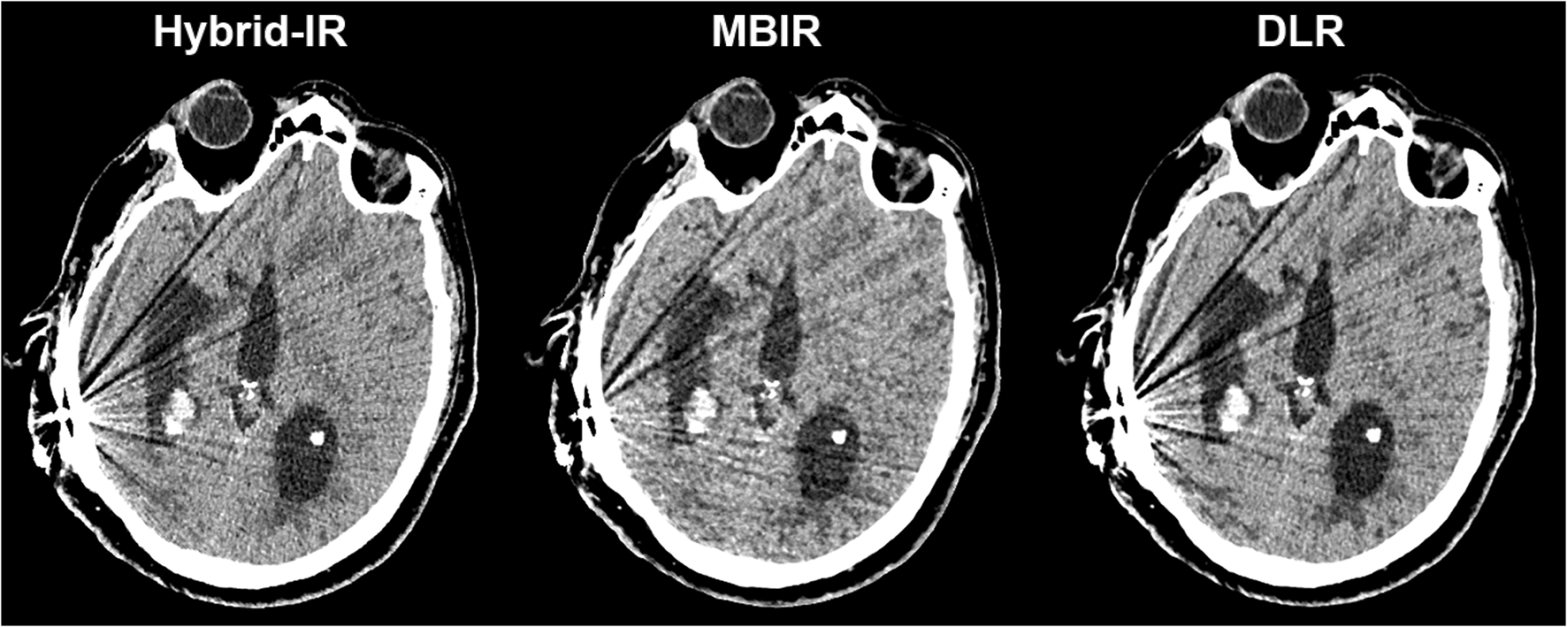
Example slice of brain acquisition with artefacts due to valve of ventriculoperitoneal shunt as presented by the different reconstruction algorithms

Artefacts are common on non-contrast cerebral CT due to beam hardening (e.g., in the posterior fossa) or due to the presence of foreign bodies. Additional noise measurements in areas prone to artefacts, such as in the fourth ventricle, did not reveal different results to those in the CSF within the lateral ventricle. In addition, subgroup analysis of the scores given in cases of patients with foreign bodies present did not show any significant differences in the observer preferences than those found for the entire case set. The artefacts due to a foreign body were found to be comparable across the different image reconstruction algorithms, as illustrated by Fig. 5.

Different acquisition techniques were used in this study. Most acquisitions (35) were made using a helical scan technique with a CTDI_vol_ of 52.7 mGy. Another 10 acquisitions were in sequential mode with a lower CTDI of 43.2 mGy. This study found significant higher image quality characteristics for the sequential scan technique. Analysis showed a non-significant interaction between acquisition technique and reconstruction method, suggesting that the statistically significant change in image quality characteristics between the two acquisition techniques is equivalent for all reconstruction techniques. Other studies found that overall image quality characteristics of helical and sequential cerebral scans are comparable, although small differences were found [16, 17]. A study on chest CT, using a CT system comparable to the one used in this study, found that sequential acquisitions that used about 10% lower dose than that used in helical scans resulted in the same image quality [18]. To investigate this effect in brain for this CT system, more research is needed.

Our study has limitations. First, only thin slice reconstructions (0.5 mm) were evaluated, but the effect of DLR on thicker slices is expected to be similar, although the noise reduction might be less pronounced since thicker slices are inherently less noisy in all reconstruction methods. Second, noise was only measured in terms of standard deviation. Although this parameter is broadly used to summarise noise, it does not incorporate the different noise textures for each reconstruction algorithm [11]. However, the noise texture is an important factor in the perception of the natural appearance [19] and this image quality characteristic is judged non-significantly different for Hybrid-IR and DLR, while DLR is preferred compared to MBIR. Finally, we only examined the preference for certain image quality characteristics and we did not incorporate a detection task evaluating the diagnostic accuracy for intracranial pathology. To determine if there is a real clinical benefit with DLR, a follow-up study incorporating detection and characterisation performance of lesions is warranted.

In conclusion, this study shows that with a slightly increased reconstruction time, DLR results in lower noise and improved tissue differentiation compared to Hybrid-IR. Image quality of MBIR is significantly lower compared to DLR with much longer reconstruction times.

## Supplementary Information

ESM 1 (PDF 345 KB)

## Abbreviations

CSF: Cerebro-spinal fluid
CTDI: Computed tomography dose index
DLR: Deep learning reconstruction
FBP: Filtered back-projection
GLM: General linear method
HU: Hounsfield unit
Hybrid-IR: Hybrid-iterative reconstruction
MBIR: Model-based iterative reconstruction
MDCT: Multi-detector CT
NCCT: Non-contrast CT
SD: Standard deviation
SDNR: Signal-difference-to-noise ratio
