# Supplementary figures and images for: Deep learning–based reconstruction may improve non-contrast cerebral CT imaging compared to other current reconstruction algorithms
Source: Eur Radiol. 2021 Mar 10;31(8):5498–506. doi: 10.1007/s00330-020-07668-x (PMC8270865; doi:10.1007/s00330-020-07668-x)

Figure S1

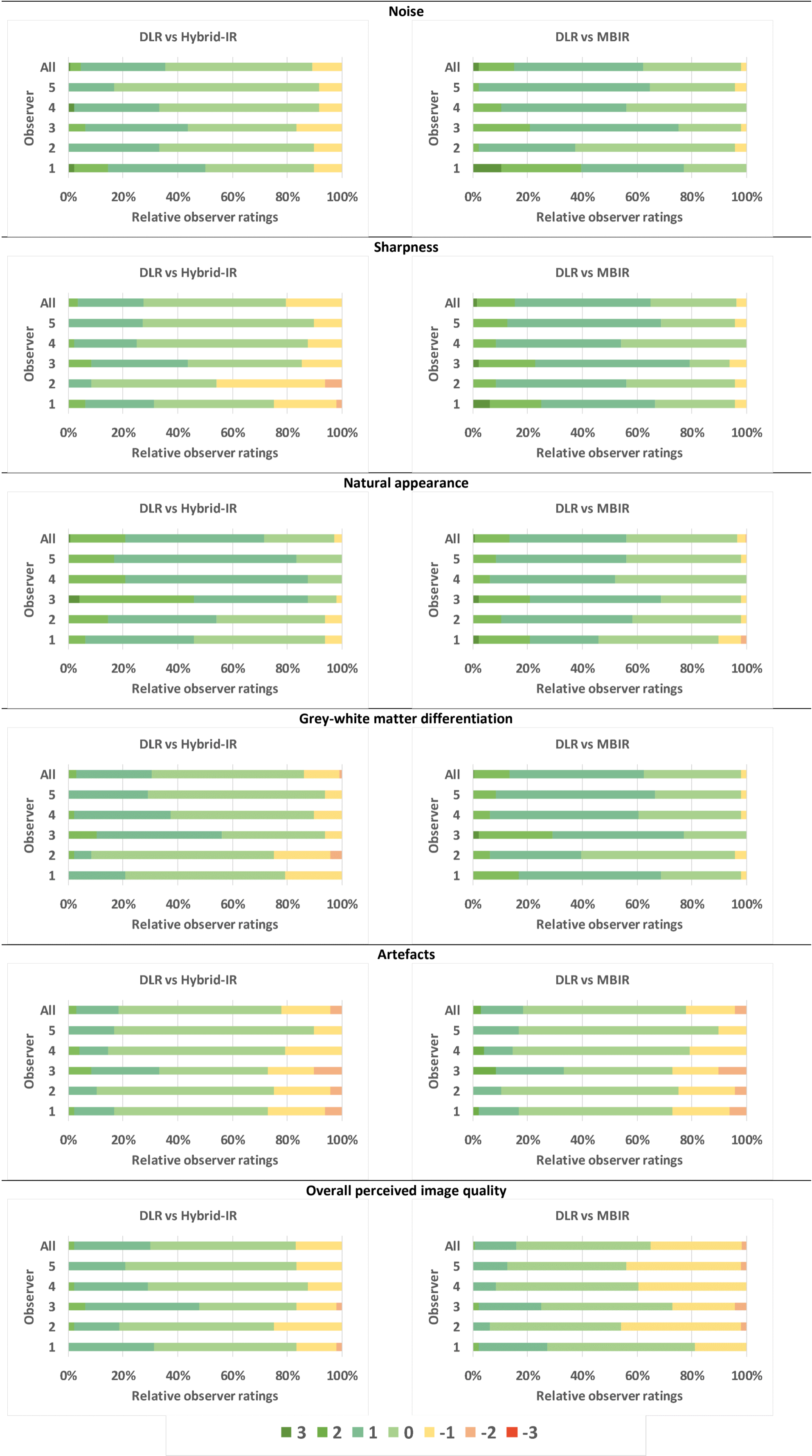

Supplement: Supplementary file 1 — (PDF 345 KB) [file 330_2020_7668_MOESM1_ESM.pdf]
